# Direct Behavioral Evidence for Retronasal Olfaction in Rats

**DOI:** 10.1371/journal.pone.0044781

**Published:** 2012-09-06

**Authors:** Shree Hari Gautam, Justus V. Verhagen

**Affiliations:** 1 The John B. Pierce Laboratory, New Haven, Connecticut, United States of America; 2 Department of Neurobiology, Yale University School of Medicine, New Haven, Connecticut, United States of America; MPI f. med. Research, Germany

## Abstract

The neuroscience of flavor perception is becoming increasingly important to understand abnormal feeding behaviors and associated chronic diseases such as obesity. Yet, flavor research has mainly depended on human subjects due to the lack of an animal model. A crucial step towards establishing an animal model of flavor research is to determine whether the animal uses the retronasal mode of olfaction, an essential element of flavor perception. We designed a go- no go behavioral task to test the rat's ability to detect and discriminate retronasal odorants. In this paradigm, tasteless aqueous solutions of odorants were licked by water-restricted head-fixed rats from a lick spout. Orthonasal contamination was avoided by employing a combination of a vacuum around the lick-spout and blowing clean air toward the nose. Flow models support the effectiveness of both approaches. The licked odorants were successfully discriminated by rats. Moreover, the tasteless odorant amyl acetate was reliably discriminated against pure distilled water in a concentration-dependent manner. The results from this retronasal odor discrimination task suggest that rats are capable of smelling retronasally. This direct behavioral evidence establishes the rat as a useful animal model for flavor research.

## Introduction

Abnormal feeding behaviors and associated chronic diseases such as obesity are becoming increasingly alarming global health issues [1]. Compulsive eating behavior, unusual preference or aversion to certain foods, as well as certain neurological problems may be directly or indirectly linked to individual’s perception of flavor. So far, neurobehavioral studies of the perception of flavor have mainly depended on human subjects due to the lack of an animal model [2], [3]. The use of humans is otherwise ideal, except that it precludes (1) the necessary invasive experimental procedures and (2) proper control of flavor experience required to explore the mechanisms underlying the perception of flavor. To expand investigations on the neural bases of perception of flavor in this study, we show evidence that the rat is a valid animal model for flavor research.

The flavor of a food generally refers to a unified multimodal percept of retronasal odor, taste and somatosensation [4]. Of these, retronasal (rather than orthonasal) odor is an important element of flavor [5], [6]. Volatiles released from the food or drink inside the mouth form the retronasal odor as they travel to the olfactory epithelium in the nasal cavity via nasopharynx. The ability of the food volatiles to act as retronasal odor may thus be affected by how efficiently the food volatiles can travel from the back of the oral cavity to the nasal cavity.

This issue becomes particularly interesting in the context of the uses of the rat as an animal model of flavor research, because rodent anatomy of the pharyngeal area is slightly different from that of adult humans. In adult humans (as well as dogs and cats [7]) the nasal passage opens widely into the oropharynx, which is also a common pathway for the food and drink before it enters into the esophagus. Such anatomical adaptation in humans allows easy transfer of food volatiles from the oral cavity into the nasal passage, particularly when the exhaled air actually passes through the oropharynx carrying along the food volatiles.

**Figure 1:**
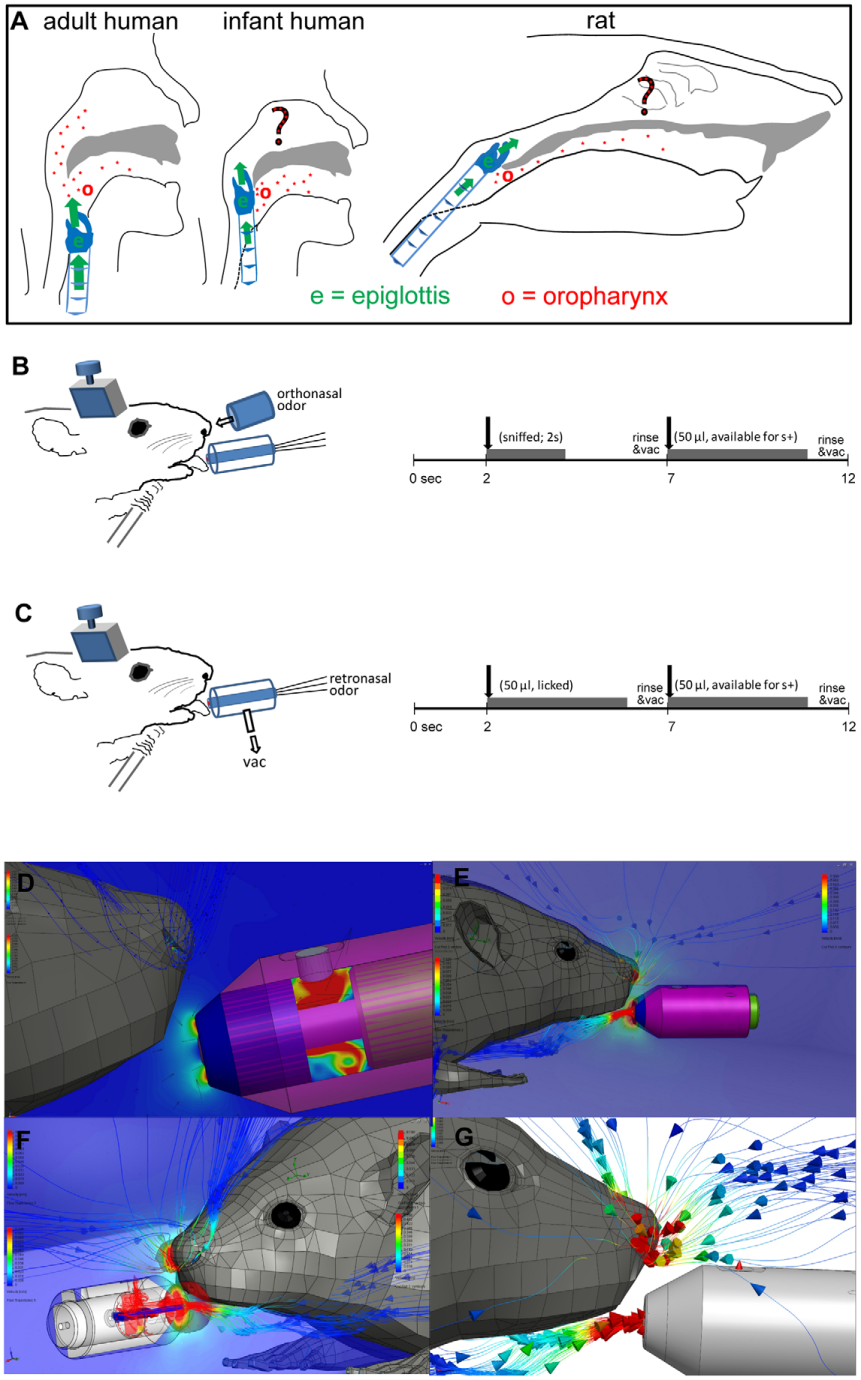
Hypothesis and retronasal-specific setup. A: Schematic diagram of obligate nasal breathing. Both rats and human infants are “obligate nasal breathers” where exhaled air from the epiglottis (green) does not effectively pass over the oropharynx (red). Moreover, the raised epiglottis may obstruct oro-nasal odorant passage (red stars). The question mark indicates that prior to this work it had not been directly tested if retronasal smell occurs in rats and infants. In humans the epiglottis descends around the fifth month of age. Such developmental decent does not occur in rodents. **B and C: Experimental set up.** Schematics of an animal performing go no-go orthonasal (**B**) or retronasal (**C**) odor discrimination task (left) and the time course of a single orthonasal trial (right). Orthonasal odorants (2% saturated vapor) were released in front of the nose by an olfactometer (**B**). Retronasal odorants dissolved in water were delivered (50 µl, after a lick) at the lick spout by a gustometer (**C**). Irrespective of the odor source, the rat could obtain water by licking the lick spout after sampling (by sniffing, **B**, or licking, **C**) an S+ odor. Vac: a vacuum tube sucking air (5 L/min) from around the lick spout to prevent orthonasal exposure. **D–G: CAD flow models suggest rats were unable to sniff the lickspout.** D: flow lines and flow velocity cut plot (i.e. a color coded flow rate along the median plane, see text; color bars: blue-red  = 0–3 m/s) at maximum reported sniff flow rate (1.8*10^−5^ m^3^/s). Lateral view. E: same at twice the maximum reported sniff flow rate. F: as in E, but now entire flow (3.6*10^−5^ m^3^/s) through the right naris (a 4-fold over-estimation). Isosurface plots (i.e. the “balloons” around the spout and the right naris) indicate volumes with at least 0.1 m/s flow rate. Cut plot color scale: blue  = 0 m/s, red  = 0.5 m/s. G is an enlargement of Fig. 1F minus the cut plot for clarity. Flow line color bars: blue  = 0 m/s, red  = 0.1 m/s.

However, in so-called ‘obligate nasal breathers’ such as rodents (as well as rabbits, horses and even human infants) the upper air passage actually meets the slightly elevated epiglottis in the nasopharyngeal area, thereby not sharing a common pathway with the food in the oropharynx [8] (**Fig. 1A**). This anatomy may have several biological advantages to the animal, such as being able to keep eating/swallowing without pausing to sniff for potential predators [8]. A potential limitation of this anatomy is that the expired air does not actually pass through the oropharynx (**Fig. 1A**), which may reduce the efficiency of retronasal delivery of the food volatiles into the nasal cavity. Whether the passage of expired air through the oropharynx is critical for the delivery of the retronasal odors is currently unknown. What is known in humans, is that food volatiles can travel retronasally to the olfactory epithelium effectively even during mastication and swallowing [9]. The same might be true for other animals including rats.

We and others have previously reported indirect evidence for the rat's use of the retronasal olfaction [10], [11], [12], [13], [14]. Steady-state air-flow models of the nasal cavity also support relevance of retronasal olfaction in rats, even though it seems less efficient than orthonasal olfaction and in humans [15], [16]. It has also been demonstrated *in vivo* that odorant stimulation of rat olfactory receptor neurons (ORNs) is possible retronasally, with more efficient delivery of lipophilic odorants compared to hydrophilic ones [17]. However, a behaving rat's ability to use the retronasal mode of olfaction has never been tested directly. Here we use a direct behavioral approach to test the rat's ability to detect and discriminate retronasal odors that were presented orally in aqueous solutions. We found that rats indeed can discriminate tasteless odors retronasally. This finding helps to understand flavor processing in human infants and non-primate mammals, most of which are obligate nasal breathers, and further establishes the rat as a useful animal model for flavor research.

## Methods

### Subjects

Long-Evans female rats weighing 180–200 g were purchased from Charles River laboratories Inc. (New York, USA) and housed individually in an environment of controlled humidity (60%) and temperature (23°C). The vivarium was set with 12-h light-dark cycles and all the behavioral training and experiments were carried out in the light phase. Food was available *ad libitum* except during testing. Rats began water deprivation at least 1 week post-surgery, and 3–4 days prior to behavioral training. For water deprivation, rats were maintained at 10–15% below baseline bodyweight. During testing and training sessions (see below), rats received approximately 3–6 ml water, following which they were given 20–30 min *ad libitum* water access in their home cage. Data acquired from six rats are presented here.

### Ethics Statement

All the animals were treated according to the guidelines established by the U. S. National Institutes of Health (1986), and the experimental protocols were approved by the Institutional Animal Care and Use Committee of the John B. Pierce Laboratory (Protocol 120).

### Surgical procedures

A custom made head-restraining stainless steel cap (head-cap, roughly 12×12×12 mm) was implanted surgically on the top of the animal's skull under Isoflurane (1.5–3.5%) anesthesia. The surgery was performed aseptically and local anesthetic (2% Lidocaine, Hospira Inc., Lake Forest, IL) was applied at all incisions. Buprenex (10 μg/kg IM, Reckitt Benckiser Healthcare, Richmond, VA) was also provided as an analgesic. Throughout the surgery the rat's core body temperature was maintained at 37°C with a thermostatically controlled heating pad (Omega Engineering Inc, Stamford, CT). The restraint head-bolt was positioned on top of the skull and secured with dental cement anchored by six skull screws (0–80×1/8′′). Following surgery, animals were supplied with *ad libitum* water containing Ibuprofen (15 mg/kg) for 3 days, and given antibiotic (Baytril, 3 mg/kg i.p.). Animals were carefully monitored throughout their surgical recovery.

### Behavioral Training

Behavioral training for the go-no go odor discrimination task was performed as reported previously [18]. Animals were trained to accept head fixation and to perform lick-no lick odor discriminations using water restriction for motivation. Briefly, one to two weeks after the initial surgery, rats were water-deprived and handled daily. Rats were habituated to head restraint by gradually increasing the duration of restraint and by providing water rewards through a lick spout. When the interval between water rewards reached approximately 20 s, rats were made to discriminate one rewarded odorant (S+) from one unrewarded odorant (S−) by licking or not licking the lick spout. All training was performed in a custom restraint chamber (approx. 11′′ long ×3′′ wide ×3′′ tall, inside) made of transparent acrylic and open on the front and the back so that the animal could enter from one end and come out from the other while not head-fixed. A steel tab with a through hole extended horizontally from the roof of the restraint chamber, allowed quick restraint of the animal by its head bolt. A cross-shaped profile on top of the head-cap fitted tightly with its inverse profile in the tab, prevented head movement and allowed repeatable positioning of the animal. A lick-spout extending from the lick-manifold of the 8-channel gustometer assembly and surrounded by a concentric vacuum tube was positioned in front of the animal's mouth for water reward and retronasal stimulus delivery (**Figure 1B–C**). The inner surfaces of the chamber were cleaned with moist paper towel between each behavioral session. A bar placed in front of the animal served as a paw-rest and helped to prevent false licks from happening.

After the initial habituation to head fixation, rats were restrained daily for a single session lasting 30–90 min (100–300 trials). Rats were rewarded with approximately 50 μl water for correct licks to the S+. Rats were rewarded when they licked during or up to 4 sec after the start of the presentation of the S+ (hit). False alarms (licks to S−) initiated a punishment of 10–20 sec added to the next inter-trial interval (iti). Rewards were not given for not licking to the S−. Odorants were presented in pseudo-random fashion (50/50 S+/S−, less than 4 consecutive trials of same valence). The total number of correct responses (S+ lick or S− no lick) was compared to the total trials for an index of behavior performance; therefore, a performance index of 40–60% was considered chance performance and a score of 100% was perfect discriminatory behavior. Signs of distress (vocalizations, defecation or failure to perform the task) resulted in cessation of the session and release of the animal from restraint. Odorant delivery, reward delivery, sniffing measurements and monitoring of performance was achieved through custom software written in LabVIEW (National Instruments, Austin, TX).

**Figure 2:**
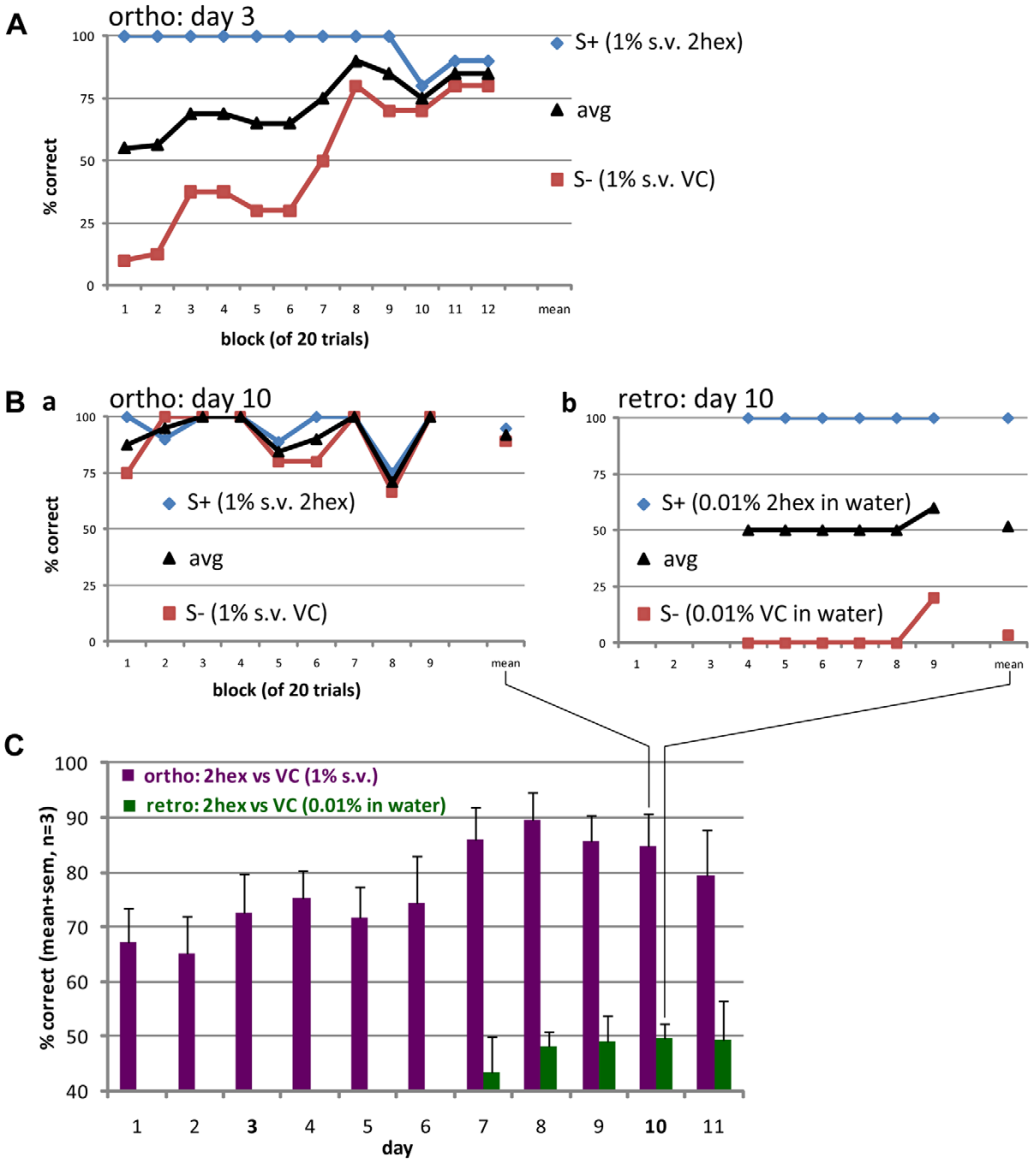
Learning an orthonasal odor discrimination task did not help learning discrimination of the same odors retronasally. **A.** Orthonasal odor discrimination by an average performing rat on the third day of training. Each block consisted of 20 trials separated by 10 s (ITI) with an additional 10–15 s (punishment) for an incorrect lick. S+ = 1% (s.v.) 2-hexanone, 2hex; S− = 1% (s.v.) vinyl cyclohexane, VC; avg  =  mean of S+ and S−. **B.** Orthonasal (left) and retronasal (right, from fourth block onwards) odor discrimination by a rat on day 10. Note that even though the rat was performing well for orthonasal odors (**a**), she failed to discriminate the same odors retronasally (**b**). Orthonasal odors were the same as in **A**. Retronasal odors were as follows: S+ = 0.01% 2-hexanone in water, 2hex; S− = 0.01% vinyl cyclohexane in water. **C**. Average daily performance of 3 rats for ortho- and retronasal discrimination of 2-hexanone vs. vinyl cyclohexane. Rats learned to discriminate the orthonasal odors as early as day 3 of the training, but failed to do so for orally ingested/retronasal odors.

One batch of animals (data shown in **Fig 2**) started discriminating odors orthonasally (**Fig. 1B**), followed after several days by discriminating odors retronasally (**Fig 1C**). In the orthonasal paradigm the rat had to sniff the air flow diluted odorant and decide whether to lick the spout to receive water reward (in case of vapor-phase S+ odor, **Fig. 1B**). For the later retronasal trials the rats had to lick the lick spout to sample the aqueous odor, after which they could lick the same lick spout again to obtain a water reward (in case of S+ aqueous odor, **Fig. 1C**).

**Figure 3:**
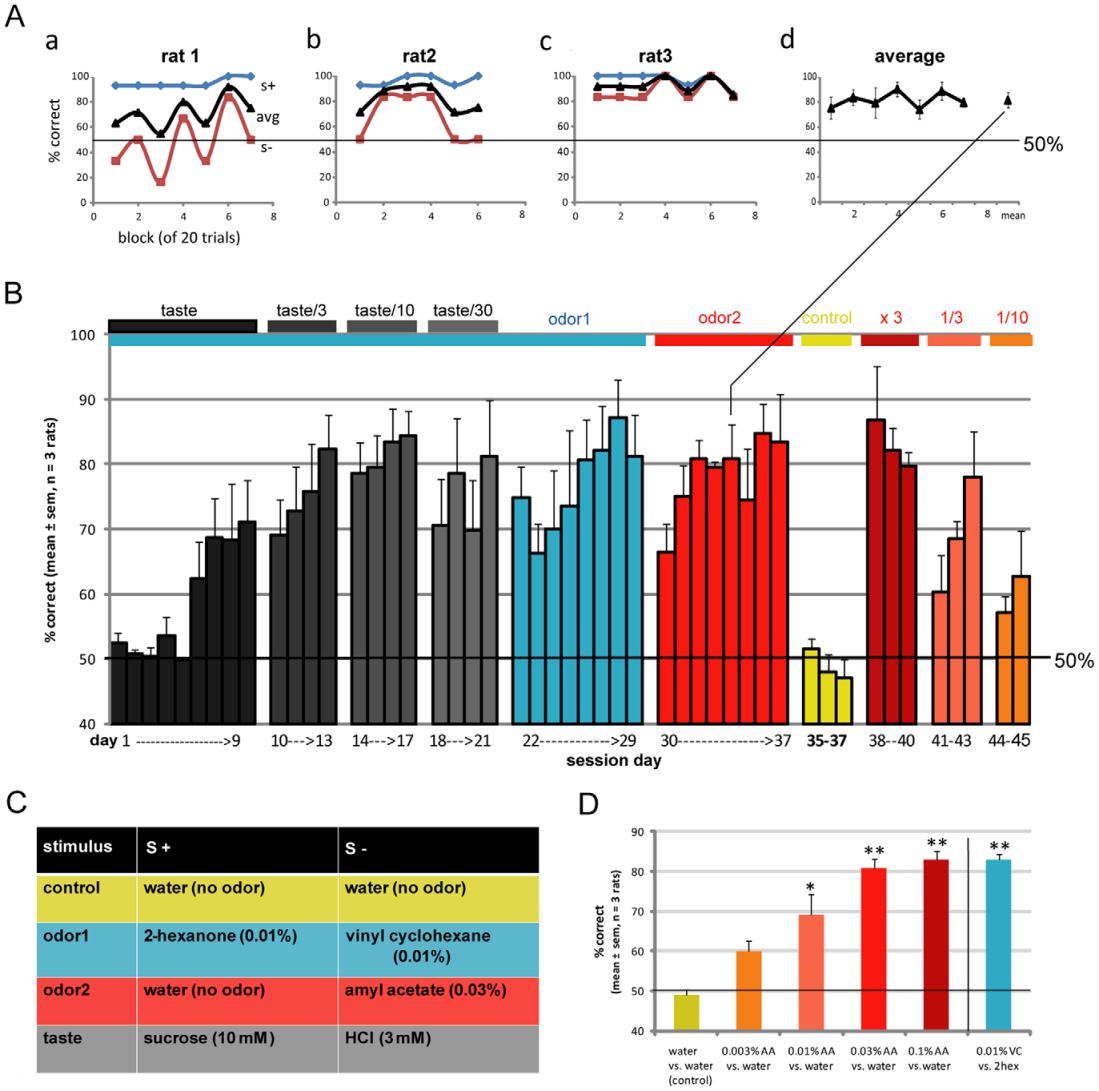
Successful taste-guided learning of go-no go retronasal odor discrimination task by rats. A . An example of a retronasal daily session for the three rats (**a–c**) and their average (**d**) (day 34 of **B**). S+ = 0.03% amyl acetate in water, S− =  water. **B**. Successful performance of go-no go retronasal odor discrimination by 3 head-fixed rats. Aqueous solutions of odorants were initially combined with tastants in order to enhance shaping (day 1–9). Tastant were gradually removed (day 10–21), leaving only retronasal odorants (day 22–29). Subsequently the same animals learned to discriminate a different odorant against water in a concentration-dependent manner (day 30–45, with cue control day 35–37). **C**. Color legend for **B** & **D**. **D**. Overall performance of the three rats. Tasteless retronasal amyl acetate was convincingly discriminated against water in a dose-dependent manner. ANOVA on concentrations across rats, F_(4,10)_ = 16.8, P<0.0005.** t-test: above control, p<0.005; * t-test: above control, p<0.05.

The first batch of animals as well as a second batch performed an additional task of gustatory discrimination with gradual removal of the tastant. This is the exact same paradigm as the retronasal paradigm: a sample lick followed by a reward lick (for data see **Fig. 3** **–** **5**).

**Figure 4:**
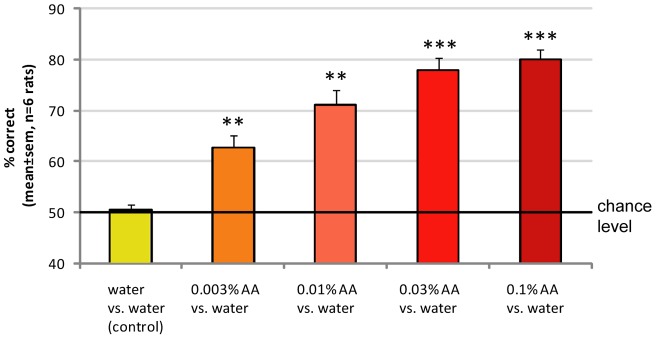
Overall performance of all six rats tested. All 6 animals learned to perform the task well above chance level, and convincingly discriminated tasteless amyl acetate against water in a concentration-dependent manner. ANOVA on concentrations across rats, F_(4, 25)_ = 22.0, P<10^−7^.*** t-test: above control, p<10^−4^; ** t-test: above control, p<0.005.

**Figure 5:**
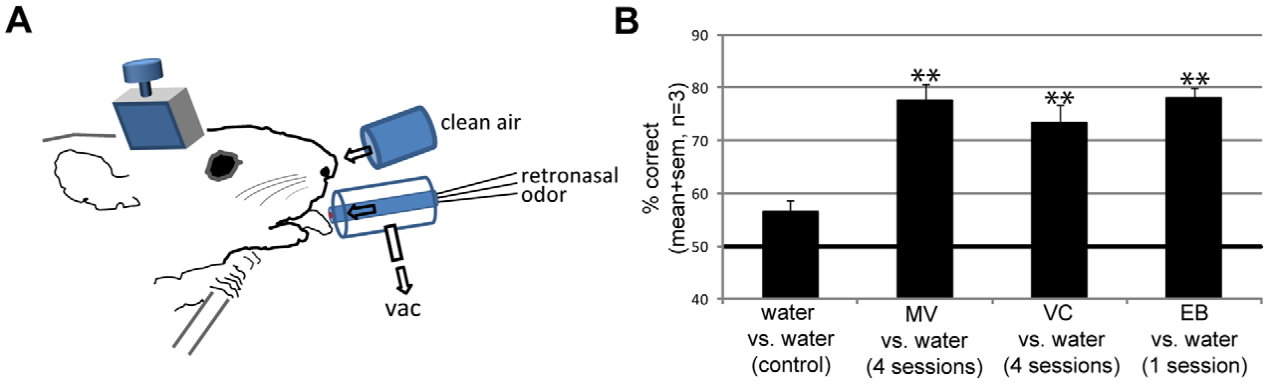
Confirmation of strictly retronasal detection of odorants. **A–B:** To ensure that rats were only depending on licked retronasal odors, we added a constant flow of clean air (5 L/min) targeting the nose. Flow models again confirmed no possible flow from lickspout to nose. Overall performance of 3 rats under these conditions averaged over 4 sessions per odor is shown (EB: 1 session). Note that rats were still able to discriminate tasteless odorants against distilled water, and their performance was not affected significantly. ANOVA on stimuli across sessions, F_(3, 8)_ = 43.5, P<0.0001. *** t-test: above control, p<10^−5^; ** t-test: above control, p<0.005.

### Olfactometry

The 16-channel olfactometer assembly for the orthonasal odor delivery was made up of Teflon odorant vials, Teflon manifolds, and Teflon tubing and connectors. Odorants were diluted from saturated vapor of pure liquid odorant using mass flow controllers (1–10 ml/min; Aalborg). Nitrogen was used as the saturated vapor carrier to prevent odorant oxidation and diluted in a stream of filtered medical grade air flowing at 500 ml/min. Linearity and stability of the olfactometer were verified with a photoionization detector (MiniRae 2000, RAE Systems Inc., San Jose, CA). An odorant delivery tube, positioned ∼6 mm from the animal's nose, assured rapid onset and offset of the odorant (**Fig. 1B**). Odorant duration was 2 sec. Retronasal odors were delivered using gustometry.

### Gustometry

The 8-channel gustometer assembly served for the delivery of retronasal aqueous odor stimuli, taste stimuli, as well as the water reward through dedicated channels for each solution. The gustometer consisted of 1 or 5 L glass bottles, Teflon tubing, connectors and manifold. One of the channels was used for rinsing the lick manifold after each stimulus delivery; another one was used to vacuum the manifold during and after rinsing. Odorants, with or without tastants, were dissolved in distilled water. Nitrogen (2.5 psi) was used to push the liquid through the solenoid valves and to prevent oxidation of the odorants in water. Each valve's activation time was calibrated separately for the delivery of a measured amount (50 µl) of the liquid. A continuous vacuum sucking air (5 L/min) from around the lick spout was used to prevent orthonasal contamination, confirmed by 3D flow models (**Fig. 1C–G**).

### Novel lickometer

Licking was recorded using a novel optical approach with full electrical isolation that is especially suited for electrophysiological experiments. We inserted a dual optical fiber assembly contained in a stainless steel tube (NF-DM03, OPTEX FA CO., LTD., Japan) into the lumen of our gustometer lick manifold (see Supplementary Figure 1 in [13]), with its tip flush with the tip of the lick spout. The two optical fibers were connected to a fiber optic reflection measurement circuit with built-in thresholding (D2RF-TN, Digital Fiber Amplifier, OPTEX FA CO., LTD., Japan). One of its connector sends red light, while the other detects its returned brightness. All settings on the D2RF-TN were left as default, except that we used the standard response speed, no sensitivity correction and set the threshold to ∼“700”. This approach is similar to that reported by Schoenbaum et al. [19], which used it to detect licking from a well, rather than from a lick spout. A lick was registered when the light circuit was triggered by a sufficient amount of light reflected from the tip of the tongue. We validated this method by concomitantly recording licking with a standard capacitance circuit and video analysis of top and side views of several head-fixed rats. Like the capacitive circuit our optical approach correctly detected true licks, but unlike the electrical sensor it did not incorrectly count events when the rat touched the spout with its paws. Moreover, our approach entirely avoids electrical lick artifacts.

### Flow modeling of retronasal setup

Rats can sniff up to 19 ml/s maximally (Youngentob et al.), ¼ of our lick-spout vacuum flow rate of 80 ml/s. Nevertheless, to ensure rats could not detect odorants licked from the lick spout we modeled the setup in 3D in SolidWorks 2011–2012 and Flow Simulation 2011. A rat (www.3dcadbrowser.com, model 4621) was placed centered in front of the lick spout closely (mm-level accuracy) matching the real setup. It was meshed with 917,181 fluid cells and 207,512 partial cells in a 1 m^3^ box (**Fig. 1** **D–G**). A finer mesh was used for the rat (Local Initial Mesh level 5) and lick spout (Local Initial Mesh level 4) than elsewhere (otherwise automatically optimized). Either one or both nares were modeled as an Outlet Volume Flow (1.8*10^−5^ m^3^/s or 3.6*10^−5^ m^3^/s, the maximum, respectively twice the maximum total sniff flow rate reported by Youngentob et al. [20]). The box boundary was set as Static Pressure (101,325 Pa) and temperature at 293 K. The lickspout vacuum tube connector was modeled as an Outlet Volume Flow (8*10^−5^ m^3^/s). Solutions of steady state flow, the global goal being the average velocity, were reached after ∼800 iterations which each required ∼8 hrs. time on a quad core PC.

### Odorants and tastants

Variety of monomolecular odorants was selected based on their solubility and previous use in olfactory research involving rats. All odorants (amyl acetate, methyl valerate, ethyl butyrate, vinyl cyclohexane and 2-hexanone) and tastants (sucrose and HCl) were reagent grade and purchased from Sigma (Sigma-Aldrich, St. Louis, MO). Odorants were stored in the dark under nitrogen.

### Data analysis

First, the percentage of correct licks (for S+) and the correct rejects (for S−) within each block of 20 trials were averaged. Then the percent correct for each block in a session were averaged to obtain a daily % correct per animal. All the blocks of a daily session were included in the analysis. The final % correct per stimulus condition was obtained by averaging daily % correct across animals. These data were further analyzed by analysis of variance (ANOVA) (test stimulus as main factor) and planned *t*-tests. Averages are reported ± standard error of the mean (standard deviation/√n). Alpha level was set at 0.05.

## Results

### Prior orthonasal task acquisition does not improve retronasal task acquisition of the same odorant

Before introducing retronasal odors we first tested if our rats could learn to discriminate orthonasal odors (**Fig. 1B**), and if a rat's ability to discriminate orthonasal odors would help them to learn discriminating similar odors presented retronasally. In this orthonasal paradigm the rat had to sniff the air flow diluted odorant and decide whether to lick the spout to receive water reward (in case of vapor-phase S+ odor, **Fig. 1B**). As shown in **Figure 2A** our head-fixed rats were able to discriminate between 2-hexanone and vinyl cyclohexane (both at 1% s. v., sniffed, presented via olfactormeter's odor tube) as early as the third day of the training. Typically, during task acquisition the performance would increase gradually during the course of a daily session. When the average daily performance for the orthonasal task reached 80% or above, half of the trials within each block contained the aqueous solution of the same set of odorants (0.01% in distilled water) presented orally, instead of orthonasally. For clarity, here rats had to lick the lick spout to sample the aqueous odor, after which they could lick the same lick spout again to obtain a water reward (in case of S+ aqueous odor, **Fig. 1C**). At the start of the daily session only orthonasal odors were presented. Retronasal trials were begun only after a few blocks of consistently well performed orthonasal trials (**Figure 2B**). Rats successfully performing the orthonasal odor discrimination task could not readily learn to discriminate the same set of odorants when licked (**Figure 2B & C**). There was no sign of retronasal task acquisition even during the second half of the daily session after 5 days of the training. The average daily performance of the 3 rats for ortho- and retronasal odor discrimination of 2-hexanone vs. vinyl cyclohexane are summarized in **Figure 2C**. Moreover, after day 11 (**Fig. 2C**) and before day1 (**Fig. 3B**) we tested the 4 rats with only retronasal stimuli for 4 days. There was no evidence of learning: the performance was still random (53.9±2.0%, mean±s.d) on the 4th day. Rats learned to discriminate orthonasal odors as early as day 3 of the training, but failed to do so for orally ingested/retronasal odors.

To ensure that odorants from the lick spout could not have been sniffed we investigated the source of air flow entering the nares in the 3D flow model which accurately represented the setup. The resolved flow lines are most clearly depicted in **Fig. 1G** where 100 flow lines enter the left naris and 200 the lick spout vacuum. It is clear that none of the flow lines originated from the lick spout, as they are completely segregated between the nose and lick spout. Sniffed air instead originated from above and in front of the rat. Furthermore, the vacuum recruited air surrounding the rat's mouth (**Fig. 1E–G**), implying that the tongue becomes surrounded by the vacuum airflow during licking. This is a conservative conclusion in that the solution of **Fig. 1F–G** used twice the maximum reported sniff flow rate and this was entirely based on inhalation via only the right naris. Less extreme cases (**Fig. 1D–E**) yielded the identical conclusion that the vacuum prevented orthonasal smell or the retronasal stimulus. Note how the cut plot in **Fig. 1D**, which plots the air velocity using a color map (blue  = 0 m/s, red  = 3 m/s) along the median plane that “cuts” through the middle of the rat and the lick spout vacuum, finely captures the flow peaking and exiting from the top of the concentric vacuum tube as well as the high velocity flow surrounding the lick spout. Cut plots are shown in all but Fig 1G, showing that flow rates are low (hence the blue back ground) everywhere except at the nares and vacuum entry of the lick spout (red).

### Initial taste-guidance leads to retronasal odor discrimination

As the retronasal odors naturally co-occur with taste associated with the food we next taste-guided the rats to discriminate retronasal odors by initially mixing a tastant with each odorant. The idea was to first draw the rat's attention to taste, to establish rat's ability to learn the go-no go task for taste discrimination, and then to remove the taste component gradually so that only the aqueous odorants remained for the rats to discriminate (**Figure 3A**). As shown in **Figure 3B** **,** rats learned to discriminate between 0.01% 2-hexanone (initially sweetened with 10 mM sucrose) and 0.01% vinyl cyclohexane (initially made sour with 3 mM HCl; for legend, see **Figure 3C**). Rats initially appeared to depend upon the taste perception up to 1/10^th^ of the original tastant concentration (**Figure 3B** **, day 1–17**), and when tastant concentration was further lowered (**Figure 3B** **, day 18–21**) or removed completely (**Figure 3B** **, day 22–29**), they took an average of 3 and 5 days, respectively, to reestablish performance to criterion. Both of these odorants at 0.01% concentration were tasteless to the experimenters.

### Concentration-dependent retronasal discrimination

As we could not completely rule out the presence of a gustatory or somatosensory component in the aqueous odorants mentioned above, we next used aqueous amyl acetate, which is devoid of taste and mouth-feel up to 0.1% in rats [11], as S+ vs. distilled water as S−. As can be seen in **Figure 3A** **(day 34) & B** (day 30–37) rats successfully learned to discriminate between the presence and absence of 0.03% amyl acetate in water. Between day 29 and 30 one training day was used to switch the S+ odor 1 to water (new odor 2 S+, not shown for clarity). The accuracy of discrimination of amyl acetate vs. water averaged across days in the three rats reliably varied with amyl acetate concentration (0.003%–0.1%; ANOVA on concentrations across rats, F_(4,10)_ = 16.8, P<0.0005; **Figure 3D**). The experiments were repeated with a second batch of three rats. The overall performance of all six rats tested is shown in **Figure 4**. All animals learned to perform the task well above chance level, and convincingly discriminated 2-hexanone against vinyl cyclohexane, and amyl acetate against water in a concentration-dependent manner (ANOVA on concentrations across rats, F_(4, 25)_ = 22.0, P<10^−7^). We replaced the odorant with water as our control for non-chemical cues, including auditory cues, which indeed was negative in all cases (**Figure 3B** **(day 35–37), 3D and 4**).

### Validation of strictly retronasal but not orthonasal odorant delivery

To prevent the rats from smelling the licked retronasal odorant (orthonasal contamination), our setup incorporated a vacuum around the lick spout (**Fig. 1C–G**). As rats sniff maximally at a flow rate of 19 ml/s [20] we choose the vacuum to be four times stronger (80 ml/s, 5 l/min). Flow modeling confirmed that rats could not have sniffed the retronasal odorants, even if the rat would have sniffed at twice this maximum flow rate through either both nares or even a single naris (**Fig. 1** **E–G**). Nonetheless, to further ensure that no trace of odorants escaped the suction of the constant vacuum around the lick-spout and to make sure odor was not released from the mouth to the external nares during ingestion of the odorized water, 3 rats (second batch, never exposed to orthonasal odors) performed discrimination tasks of different retronasal odorants while we added a constant flow of clean air (5 l/min) aimed at the nose, as shown schematically in **Figure 5A**. The 3D flow model of this enhanced setup showed this to be effective (data not shown). Overall performance under these conditions averaged over 4 sessions per odor (1 session for EB) is shown **Figure 5B**. Rats were still able to discriminate between water and retronasal odorants (0.01% in water), both for novel odorants (methyl valerate and ethyl butyrate) as well as a previously experienced odorant (vinyl cyclohexane, used during training) (ANOVA on stimuli across sessions, F_(3, 8)_ = 43.5, P<0.0001). Their performance was not decreased by blowing away any possible trace of orthonasal contamination. In addition, during one last session of two rats we provided tasteless 0.01% retronasal AA versus water, either with or without the 5 L/min flow. Without this flow their performance was 79.5±3.9%, similar to their performance in the presence of air flow (83.3±2.8%). Hence we conclude that the 5 L/min vacuum applied around the lick spout in our experimental set up was sufficient to remove any trace of odorants potentially released from the drop of odorized water. The additional provision of a strong flow of clean air toward the nose was not necessary to prevent orthonasal contamination of the odor.

## Discussion

### Relevance of olfactory dual modality in rats

It is well established in humans that odorants can reach the olfactory epithelium via two routes: orthonasally, when volatiles in the outside world enter the nasal cavity during sniffing, and retronasally, when volatiles released in the mouth pass into the nasal cavity via nasopharynx during eating. Rozin [4] considered this as two distinct modes of olfaction and hypothesized that the same olfactory stimulation may be perceived in two different ways depending upon whether it is coming from the mouth (oral capture), or from the external world. Several recent psychophysical [21], [22], [23], [24] and human brain imaging studies [3], [23] support this hypothesis and suggest that ortho- and retronasal delivery of the same odorant evokes distinct perceptions and patterns of neural response in the brain. Indeed, Scott and colleagues reported that olfactory epithelial responses depend greatly on whether the flow is ortho- or retronasally directed [17]. In our first experiment (**Figure 2**) rats that efficiently discriminated two different odorants orthonasally (∼90% accuracy) were not able to discriminate the same odorants when presented retronasally (∼50%, chance level). Even though the effective concentration and distribution pattern of the odorants on the olfactory epithelium by the two routes might have been significantly different [25], [26], [27], one would still expect some degree of similarity in the perceived quality of the odor and hence in the ability of the rats to discriminate the same odors retronasally. On the other hand, all the rats we tested learned to discriminate the same odorants when initially guided by taste (**Figures 3**–5). It is quite possible that the retronasal task was too confusing for the rats when both ortho- and retronasal trials were presented within a block (see **Figure 2C**).

Isolation of the olfactory retronasal stimuli from orthonasal contamination was achieved by using a vacuum around the aqueous odor providing lick spout. Selective retronasal stimulation without simultaneous gustatory and somatosensory stimulation of the oral cavity required the use of aqueous odorants at concentrations low enough to be orally neutral (“tasteless”), yet high enough to be retronasally detectable. Amyl acetate at concentrations below 0.1% in aqueous solutions is not detectable in bulbectomized rats [11], which is why we based our concentration-response study on this odorant. Thus, rats can use neither gustatory nor somatosensory cues to detect amyl acetate at concentrations at which our rats' detection performance was significantly above chance (**Fig. 4**). Interestingly, the detection threshold of retronasal amyl acetate appears to be 30 times lower (0.003%, **Figure 4**) than the highest concentration that still is orally undetectable**.** However, it should be noted that our rats licked 50 µI and Slotnick's rats 20 µl. Even if this were to yield 2.5 times lower threshold, we still used a concentration an order of magnitude below that threshold. We were unfortunately unable to perform bulbectomies in these rats, which would have been a desirable control for oral sensing of the reportedly undetectable odorant. Nevertheless we conclude it was unlikely that the rats were able to detect the amyl acetate stimulus by other means than the olfactory system, especially at the lower 2 concentrations. We have now also reported [28] that we get reliable responses in the olfactory bulb to retronasally presented odorants in anesthetized rats at physiological flow rates, providing further evidence that retronasal smell is likely a key part of the rats flavor perceptual repertoire.

### Obligate nasal breathing and ability to smell retronasally

Anatomically, a slightly elevated epiglottis opening into the nasopharynx, instead of the base of the oropharynx, makes an animal an obligatory nasal breather (**Fig. 1A**). The term obligate nasal breather is, however, somewhat misleading because it tends to imply that the animal would be unable to breathe through its mouth at all, which is not the case. There still exists a potential path for the passage of air between the nasopharynx and the oropharynx. That is why orotracheal intubation has been possible in horses [29], [30] and rodents [31], and why human infants may breathe through their mouth spontaneously or during nasal occlusion [32]. Human infants are considered to be obligate nasal breathers only until the maturational descent of epiglottis around 4–6 months of age [33].

Our demonstration of the rat's likely ability to detect and discriminate orally presented aqueous solutions of reportedly tasteless odorants in the tentative absence of orthonasal contamination also suggests the patency of the oro-nasal path for the retronasal entry of the food volatiles into the nasal cavity.

Obligate nasal breathing is often believed to be an adaptation which allows a prey animal to keep its orthonasal olfactory sensing for potential predators undisturbed while feeding/grazing [8]. This is not possible in adult humans where breathing is temporarily halted during swallowing. Indeed, the ability to swallow food/drink while breathing through the nose is the most salient feature of obligate nasal breathers [34].

Our findings thus suggest that obligate nasal breathing may not deprive the animal of the ability to smell retronasally and hence to perceive the flavor of the food being eaten.

The present study constitutes the first direct behavioral evidence for retronasal olfaction in rats. As a next step towards establishing the rat as an animal model of flavor research it would be interesting to record olfactory neural responses to retronasal stimulation during behavioral odor discrimination.
